# Pharmacology of the Adenosine A_3_ Receptor in the Vasculature and Essential Hypertension

**DOI:** 10.1371/journal.pone.0150021

**Published:** 2016-02-23

**Authors:** Ming-Fen Ho, Leanne M. Low, Roselyn B. Rose’Meyer

**Affiliations:** Heart Foundation Research Centre, School of Medical Science, Griffith University, Gold Coast, Queensland, Australia; Fraunhofer Institute for Cell Therapy and Immunology, GERMANY

## Abstract

**Background:**

Essential hypertension is considered to be a multifactorial disorder and its aetiology has yet to be clearly identified. As the adenosine receptors have a significant role in mediating vasodilation, alterations in their structures or signalling pathways may be involved in the development of hypertension. This study aimed to measure the expression of adenosine A_3_ receptors in a range of cardiovascular tissues and determine whether they could be altered with essential hypertension, and to functionally test responses to adenosine A_3_ receptor agonists in coronary blood vessels using the isolated perfused heart preparation.

**Methods:**

mRNA samples from cardiovascular tissues and a range of blood vessels were collected from 10 week old male spontaneously hypertensive rats and age-gender matched Wistar rats (n = 8). The Langendorff heart perfusion preparation was used to characterise adenosine A_3_ receptor mediated coronary vasodilation in the rat heart.

**Results:**

Adenosine A_3_ receptor agonists induced coronary vasodilation. The expression of adenosine A_3_ receptors in cardiovascular tissues was altered in a tissue-specific pattern. Specifically, down-regulation of adenosine A_3_ receptor expression occurred in hypertensive hearts, which might be associated with attenuated vasodilator responses observed in coronary vessels to adenosine A_3_ receptor agonists.

**Conclusions:**

This study demonstrated alterations in the expression of adenosine A_3_ receptors occurred in a tissue specific mode, and reduced adenosine A_3_ receptor mediated coronary vasodilation in hearts from spontaneously hypertensive rats. Our findings with regard to changes in the adenosine A_3_ receptor in hypertensive hearts suggest that adenosine A_3_ receptor might play a role in the physiopathology of essential hypertension and potentially open the way to pharmacologic manipulation of vasomotor activity by the use of adenosine A_3_ receptor agonists.

## Introduction

Essential hypertension (EH) is a major public health issue that is estimated to affect 20% of the adult population worldwide [1,2]. It is considered to be a multifactorial disorder and its aetiology has yet to be clearly identified. Adenosine is a well-established vasodilator and its receptors are widely distributed throughout the cardiovascular system [3,4]. Several studies have reported its involvement in cardiovascular disease mediated through vessel remodelling, cell proliferation, platelet aggregation and inflammatory responses [5–8]. The four adenosine receptor (ADOR) subtypes: A_1_, A_2A_, A_2B_ and A_3_ are involved in vasodilator function through different intracellular signalling pathways [9]. Adenosine is synthesized and released from vascular smooth muscle cells and cardiac fibroblasts, cardiomyocytes and endothelial cells [10]. These receptors are widely distributed throughout the body and are able to mediate a number of different functions. In the heart, the cardioprotective role of adenosine is mediated through the adenosine A_1_, A_2A_ and A_3_ receptors, all of which have been demonstrated to cause coronary vasodilation. While the adenosine A_1_ and A_2_ receptors have been extensively researched, the present study focused on the adenosine A_3_ receptor in terms of the gene expression in cardiovascular tissues and functional role in EH.

The adenosine A_3_ receptor is coupled to several types of G-proteins. The adenosine A_3_ receptor inhibits adenylyl cyclase via G_i_ protein. This receptor is also coupled to the G_q_ protein and stimulates phospholipase C and calcium mobilization. Following activation of the adenosine A_3_ receptor, the formation of phospholipase C stimulates inositol-3-phosphate and diacylglycerol production. This in turn, increases the intracellular calcium concentration and activates protein kinase C, which then interacts with K_ATP_ channels and calcium channels in sarcoplasmic reticulum to cause vasodilation [11–13].

Adenosine A_3_ receptor selective agonists have been reported to stimulate cardiac preconditioning through activation of the K_ATP_ channels and stimulation of the RhoA-phospholipase D1 signalling pathways [14]. A role for adenosine A_3_ receptor mediated vasodilation in mouse aorta has also been demonstrated with the selective adenosine A_3_ receptor agonist 2-chloro-N^6^-(3-iodobenzyl)adenosine-5'-N-methylcarboxamide (CI-IB-MECA) which induced relaxation of the vessel at very high concentrations (100μM) [15]. While there has been extensive research into its structure and function, there are still gaps in knowledge, particularly the role of adenosine A_3_ receptors in EH.

This study was designed to investigate the expression of adenosine A_3_ receptor in cardiovascular tissues and a range of blood vessels from Wistar and spontaneously hypertensive rats (SHRs)—a well-established model of hypertension [16], and to determine whether they are altered with EH. Subsequently, we determined the functional role of adenosine A_3_ receptors in the coronary blood vessels using the isolated Langendorff perfused heart preparation—a useful tool in cardiovascular and pharmacological research [17]. This study investigated the expression of adenosine A_3_ receptor in a number of tissues and characterised adenosine A_3_ receptor mediated coronary vasodilation in the rat heart using selective agents. Functional changes in adenosine A_3_ receptor populations might play a critical role in the pathology of EH.

## Methods and Materials

### Ethics statement

The study protocol complied with Australian Ethical Standards and was approved by the Griffith University Ethics Committee. Functional studies of the adenosine A_3_ receptor in the coronary blood vessels was performed using tissues from 10 week-old male spontaneously hypertensive rats (SHRs) and age-matched Wistar rats (ARC, Australia).

### Conscious rat systolic blood pressure

Conscious rat systolic blood pressure was measured using a non-invasive blood pressure (NIBP) system. The tail cuff and the pulse transducer were placed at the proximal end of the tail. The data were recorded by ML125NIBP power lab system. Rats were resting in the cage for at least 2 hours prior to measuring blood pressure in order to reduce stress-related increases in blood pressure responses. Three readings were taken before sampling.

### Sample preparation of animal tissues

Rats were housed in a room at a temperature of 23 ± 2°C, a 12 hour light-dark cycle with free access to food and water at all times. Rats were anaesthetised using pentobarbital (60mg/kg, IP). Whole blood was collected by performing a cardiac puncture procedure. Blood samples were collected into PAXgene blood RNA tubes (QIAGEN, Australia) and stored at -80°C before processing. Dissection was immediately carried out after blood sampling. Atria, left ventricle, thoracic aorta, kidney, portal vein, mesenteric vessels and retina were isolated and dissected from surrounding fat and tissues. Tissues were then frozen using liquid nitrogen and were stored at -80°C before processing.

### RNA extraction

Blood samples (2.5ml) were collected using PAXgene tubes. RNA isolation protocol followed the PAXgene blood RNA system (QIAGEN, Australia). RNA extraction protocol, as per manufacturer’s instructions, was followed. RNA samples from tissues were extracted using the RNeasy plus mini kit (QIAGEN, Australia). The yield of total RNA and the 260/280 ratio was measured using the Nanodrop^®^ ND-1000 spectrophotometer (NanoDrop Technologies, USA). cDNA was synthesised from 5μg of total RNA using random decamer primers (Geneworks, Australia) and AffinityScript^TM^ multiple temperature reverse transcriptase (Stratagene, USA).

### Real time PCR

Gene expression was measured using real time PCR. Primer sequences of internal controls and the adenosine A_3_ receptor are listed in the **S1 Appendix**. Details for reference gene stability analysis and primer efficiency analysis as described in the S1 Appendix. 18S rRNA and β-actin (ACTB) were utilised as the internal housekeeping genes for this experiment as they have been reported to be as reliable reference genes for gene expression analysis of human ADORs [18]. The PCR mixture contained 2μl of cDNA, 4μl of 5X EvaGreen^®^ qPCR Mix Plus (ROX) buffer, 1μl of each primer (5mM) and distilled water up to 20μl per reaction. Real time PCR reactions were performed in duplicate using Rotor Gene Q (QIAGEN, Australia). The 2^-ΔΔCt^ method was employed for statistical data analysis.

### Langendorff isolated heart preparation

The non re-circulating Langendorff isolated heart preparation [17] was used to investigate cardiovascular function, specifically the role of adenosine receptor induced coronary vasodilation in control and hypertensive hearts. For each preparation, the rat was anaesthetized using pentobarbitone sodium (60mg/kg, IP). Subsequently, the heart was excised and prepared using Krebs-Henseleit solution (118mmol/L NaCl, 4.7mmol/L KCl, 1.75mmol/L CaCl_2,_ 1.2mmol/L MgSO_4_, 11mmol/L glucose, 0.5mmol/L EDTA, 25mmol/L NaHCO_3_). The aorta was cannulated and the heart perfused at a flow rate to maintain 80mmHg coronary perfusion pressure with Krebs-Henseleit solution gassed with 95% O_2_ and 5% CO_2_ and kept at 37°C. Ventricular fluid accumulation was prevented by inserting a small piece of polyethylene tubing through the apex of the left ventricle to drain the cavity and maintain aortic valve patency. A water-filled latex balloon was then introduced into the left ventricle and connected to a pressure transducer (Gould P23-ID, Oxnard Ca). End-diastolic pressure was adjusted to 4-8mmHg by inflating the balloon. Ventricular pressure was monitored continuously using a MacLab data acquisition system (ADInstruments, Castle Hill, Australia). Coronary perfusion pressure was measured using a pressure transducer connected to a water filled probe inserted into the side arm of the aortic cannula and was recorded using the Maclab data acquisition system. The left ventricular pressure signal was electronically differentiated to measure dP/dt_(max)_ and dP/dt_(min)_. Hearts were allowed to equilibrate for a period of 30 minutes. During equilibration functional measurements were also taken. The adenosine A_3_ receptor was investigated in Wistar (n = 8) or hypertensive hearts (n = 8). APNEA and CL-IB-MECA (10^−9^–10^-5^M), adenosine A_3_ receptor agonists [11], concentration response curves were performed in the absence of the adenosine A_1_, A_2A_ and A_2B_ antagonist, 8-(p-sulfophenyl) theophylline (8SPT) [19]. The heart was then allowed to equilibrate and coronary perfusion pressure to recover back to 80mmHg. APNEA (a non-selective adenosine receptor agonist known to have a high affinity for the adenosine A_1_ and A_3_ receptors) or CL-IB-MECA (a selective A_3_ agonist) concentration response curves were then repeated in the presence of the nonselective adenosine A_1_ and A_2_ receptor antagonist, 8SPT (10^-5^M). This allowed the isolation of the adenosine A_3_ receptors and demonstration of its effects in the heart.

### Measurements of parameters and statistical analysis

Data collected from the concentration response curves were statistically analysed and graphed using GraphPad PRISM 5.0 for Mac. All data is presented as mean ± S.E.M, unless stated otherwise. Concentration response curves to APNEA and CL-IB-MECA were analysed using nonlinear regression, from which EC_50_ values and 95% confidence intervals were also derived. One-way or two-way ANOVA were performed on the data, followed by Tukey’s Multiple Comparison Test/Bonferroni for individual comparisons when significant effects were detected. Differences were considered significant at p<0.05. A Student’s t test was utilised to analyse whether the relative mRNA expression from the hypertensive group was statistically different to the control group values for the adenosine receptor subtypes.

## Results

As adenosine receptors have an important role in moderating vascular function, the purpose of this study was to firstly determine whether the expression of adenosine A_3_ receptors might be altered in cardiovascular tissues of SHRs, and secondly to determine whether there is pathophysiological relevance in EH. Therefore, we set out to study the differences in expression of the adenosine A_3_ receptor between SHRs and Wistar rats using samples from atria, left ventricle, blood, kidney and a range of blood vessels, and to investigate the functional role of the adenosine A_3_ receptor in modulating coronary artery vasodilation.

### The expression of adenosine A_3_ receptor was associated with essential hypertension in a tissue specific fashion

**Fig 1:**
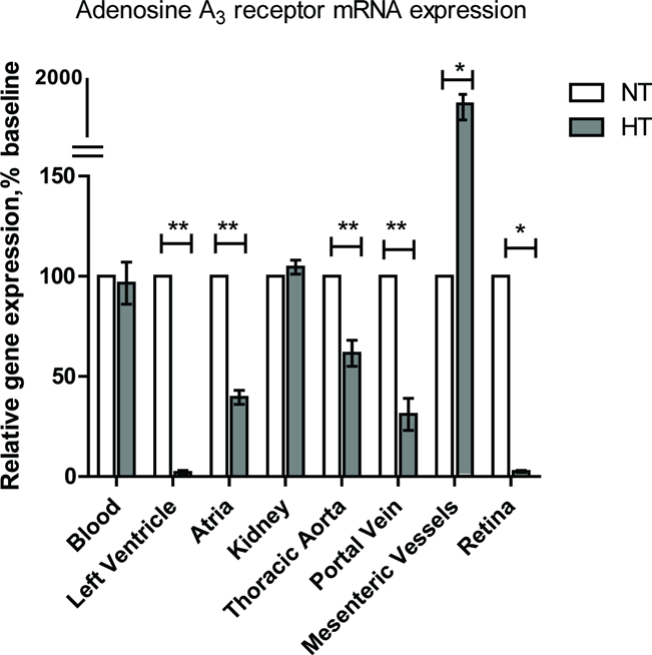
Relative mRNA expression of adenosine A_3_ receptor in a range of cardiovascular tissues. The 2^-ΔΔCt^ method was used to calculate relative mRNA expression. A Student’s *t* test was then performed as compared spontaneously hypertensive rat group (HT) with normotensive group (NT), (n = 8 for each group). Data were presented in mean±SEM. Three independent experiments were performed in duplicates. *p<0.05, **p<0.0005.

In the first step of this study, we set out to determine the mRNA expression of adenosine A_3_ receptors and its splice variant in the hypertensive and normotensive groups (SHRs *v*.*s* Wistar rats). A significant difference in the expression of the adenosine A_3_ receptor was seen between various vascular beds, see **Fig 1**. Adenosine A_3_ receptor expression was down-regulated in the left ventricle, atria, thoracic aorta and portal vein of SHRs when compared to control Wistar rats *(p* < 0.05). However, in blood and kidney samples, the expression of adenosine A_3_ receptor in the hypertensive group did not display significant difference to the age-matched control group *(p* > 0.05). As the Ct values for the adenosine A_3_ receptor splice variant in blood, left ventricle, kidney, throacic aorta, portal vein, renal arteries and mesenteric vessels were above 35, the increased variablity made quantification unreliable [20]. Consequently, the adenosine A_3_ receptor splice variant was excluded from the analysis of relative expression levels in these samples. We observed the adenosine A_3_ receptor expression was different between tissue types and with hypertension. The next series of functional experiments were used to elucidate the functional activity of the adenosine A_3_ receptor in vascular tissues with the development of EH in this model.

### Baseline measurements of rats and isolated hearts

Body weight of SHRs was lower when compared to age-matched Wistar rats (p<0.05, see **Table 1**). The systolic blood pressure in the hypertensive rat group was significantly higher than Wistar control rat group (p<0.05). Heart weights were similar (p>0.05), however when corrected for body weight, a ratio of 0.33 ± 0.01% and 0.42 ± 0.01% was observed for Wistar and SHR rats respectively, indicating that SHR hearts were significantly larger in comparison to Wistar rats (p<0.05). With respect to heart function, the basal level of coronary perfusion pressure (CPP) was similar between the two groups studied (See **Table 1**).

**Table 1:** Baseline parameters (absence of any antagonist) for control Wistar rats and spontaneously hypertensive rats (SHRs).

	Control (Wistar)	SHR
	(n = 8)	(n = 8)
**B.wt (g)**	353.0 ± 9.23	277.2 ± 6.45*
**H. wt(g)**	1.15 ± 0.03	1.15 ± 0.03
**H.wt/B.wt ratio (%)**	0.33 ± 0.01	0.42 ± 0.01*
**Systolic blood pressure (mmHg)**	108 ±16	174 ±19*
**CFR (mL/min)**	16.0 ± 0.59	16.1 ± 0.83
**CPP (mmHg)**	64 ± 3	75 ± 2

Data represented as mean ± standard error

Abbreviations: SHR, spontaneously hypertensive rats; B.wt, body weight; h.wt, heart weight; CFR, Coronary Flow Rate; CPP, Coronary Perfusion Pressure.

* denotes significant difference to control group (p<0.05)

However, the left ventricular developed pressure (LVDP), dP/dt_(max)_ and dP/dt_(min)_ were found to be significantly higher in hearts from SHRs when compared to age-matched control rats (p<0.05), see **Table 2.** In hearts from SHRs, the heart rate was approximately 14% lower than hearts for control rats (p<0.05).

**Table 2:** Baseline functional parameters for Control Wistar and spontaneously hypertensive rats in the absence and presence of 8SPT (10^-5^M).

	Wistar	Wistar	SHR	SHR
		+ 8SPT		+ 8SPT
**LVDP (mmHg)**	88 ± 4	102 ± 6	148 ± 14*	138 ± 12
**dP/dt**_**Max**_ **(mmHg sec**^**-1**^**)**	1.70 ± 0.08	1.92 ± 0.10	2.54 ± 0.23*	2.45 ± 0.22
**dP/dt**_**Min**_ **(mmHg sec**^**-1**^**)**	1.52 ± 0.08	1.80 ± 0.10	2.32 ± 0.17*	2.31 ± 0.18
**Heart Rate (bpm)**	243 ± 11.27	263.4 ± 9.13	214 ± 18.29	236.4 ± 16.37

Abbreviations: LVDP, Left Ventricular Developed Pressure; dP/dt max/min, Ventricle max/min slope

Data represented as mean± S.E.M.

* denotes significant difference to control group (p<0.05)

### Effects of APNEA, CL-IB-MECA and 8SPT on coronary vasodilation in isolated hearts

Concentrations of APNEA, a non-selective adenosine receptor agonist known to have a high affinity for the adenosine A_1_ and A_3_ receptors [11] or CL-IB-MECA, a selective A_3_ agonist [19] ranging from 10^-9^M to 10^-5^M were used in the presence and absence of the non-selective adenosine A_1_ and A_2_ receptor antagonist, 8SPT (10^-5^M), which was used to isolate the adenosine A_3_ receptor during the experimental setting. The presence of 8SPT (10^-5^M), did not influence baseline functional parameters in hearts from control or SHR rats (p>0.05).

**Fig 2:**
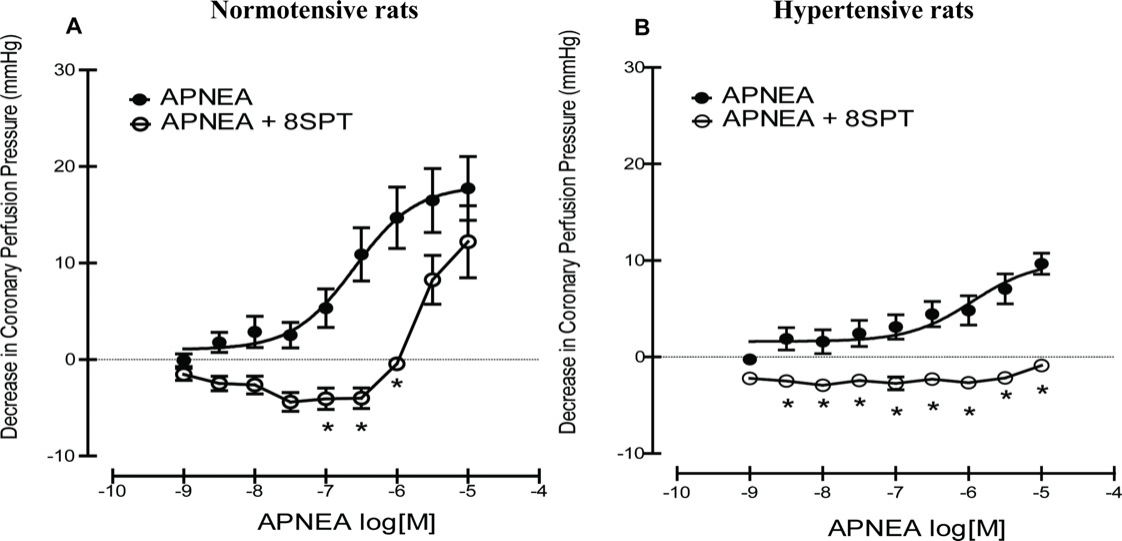
Concentration response curves showing the effects of the agonist APNEA in the absence and presence of 8SPT (10^-5^M) in (A) normotensive rats and (B) spontaneously hypertensive rats. Concentration response curves to APNEA were analysed using nonlinear regression, from which EC_50_ values and 95% confidence intervals were also derived. One-way or two-way ANOVA were performed on the data, followed by Tukey’s Multiple Comparison Test/Bonferroni for individual comparisons when significant effects were detected. *denotes statistical significant difference (p<0.05) to absence of antagonist group.

APNEA caused a concentration-dependent decrease in coronary perfusion pressure (CPP) in the absence of 8SPT in both normotensive and SHR rats (See **Fig 2**), however the response to APNEA in hypertensive hearts was depressed compared to control hearts (p<0.05). In the presence of 8SPT, which allowed the isolation of adenosine A_3_ receptor and demonstration of its mediated effects, a right-wards shift in the curve could be clearly seen in the normotensive group, indicating APNEA was able to induce coronary vasodilation mediated via the adenosine A_3_ receptor (**Fig 2A**). However, this was significantly different to hearts from SHR rats, which showed no response to APNEA in the presence of 8SPT (p<0.05), see **Fig 2B**. In addition to this, a vasoconstrictor effect was seen to precede vasodilation in the presence of 8SPT in control and hypertensive heart at lower concentrations (10^−9^–10^-6^M).

**Fig 3:**
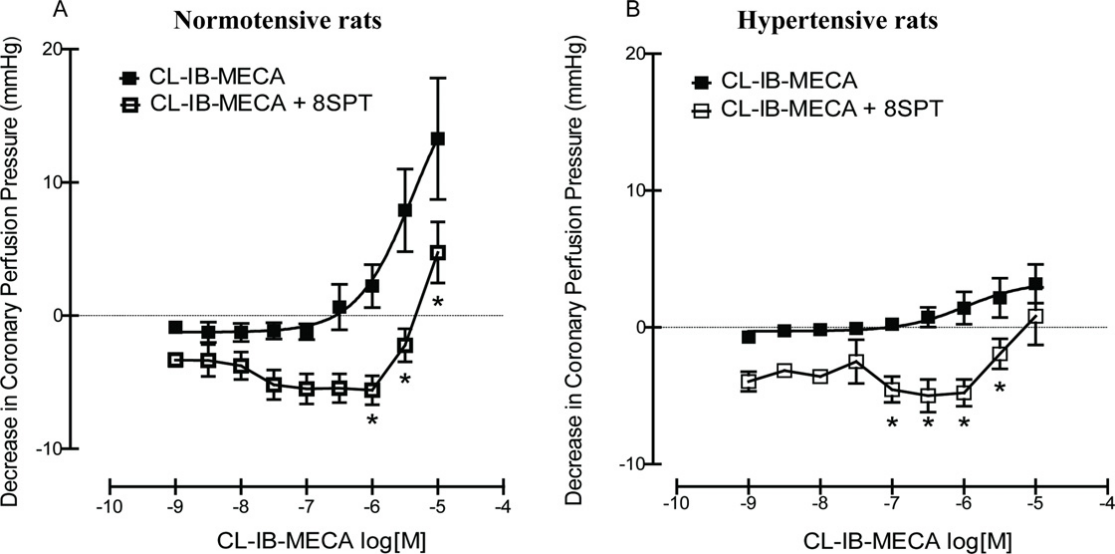
Concentration response curve showing the effects of CL-IB-MECA in the absence and presence of 8SPT (10^-5^M) in (A) normotensive rats and (B) spontaneously hypertensive rats. Concentration response curves to CL-IB-MECA were analysed using nonlinear regression, from which EC_50_ values and 95% confidence intervals were also derived. One-way or two-way ANOVA were performed on the data, followed by Tukey’s Multiple Comparison Test/Bonferroni for individual comparisons when significant effects were detected. *denotes statistical significance (p<0.05) compared to absence of antagonist group.

We performed the same experiment using CL-IB-MECA, a selective adenosine A_3_ agonist, which also showed a concentration-dependent decrease in CPP in hearts from SHR and age-matched control Wistar rats (p<0.05) (**Fig 3**). As with APNEA, there was a significant difference in concentration response curves in the absence of 8SPT in between hearts from normotensive and hypertensive rats. Specifically, hearts from SHR rats showed an attenuated response (p<0.05). Concentration curves in the presence of 8SPT (10^-5^M) caused a significant shift of the CL-IB-MECA curve in both groups studied (p<0.05) (**Fig 3**). Significant differences in APNEA and CL-IB-MECA concentration response curves on coronary perfusion pressure between control and SHR were observed (p<0.05). A decrease in the potency for APNEA was observed, whereas a slight increase in potency was seen for CL-IB-MECA. The pEC_50_ values obtained for APNEA in the presence or absence of 8SPT were significantly higher in control Wistar rats. No significant difference was observed for pEC_50_ values of CL-IB-MECA between control and SHR hearts, (See **Table 3**).

**Table 3:** pEC_50_ values for APNEA and CL-IB-MECA concentration response curves in the absence and presence of 8SPT (10^-5^M) for control (normotensive Wistar rats) and spontaneously hypertensive rats (SHRs).

Group	Control Wistar	SHRs
**APNEA**	6.60 (6.13–7.07)	5.95 (5.31–6.58)*
**APNEA + 8SPT**	5.36 (4.85–5.88)^#^	4.17 (3.86–4.49)*^#^
**CL-IB-MECA**	5.369 (4.70–6.04)	5.976 (5.04–6.91)
**CL-IB-MECA + 8SPT**	4.73 (4.54–4.92)^#^	5.10 (4.65–5.54)*^#^

Data represented as mean (lower confidence interval-upper confidence interval).

*denotes significant difference to control group (p<0.05)

# denotes significant difference to absence of antagonist group(p<0.05)

**Fig 4:**
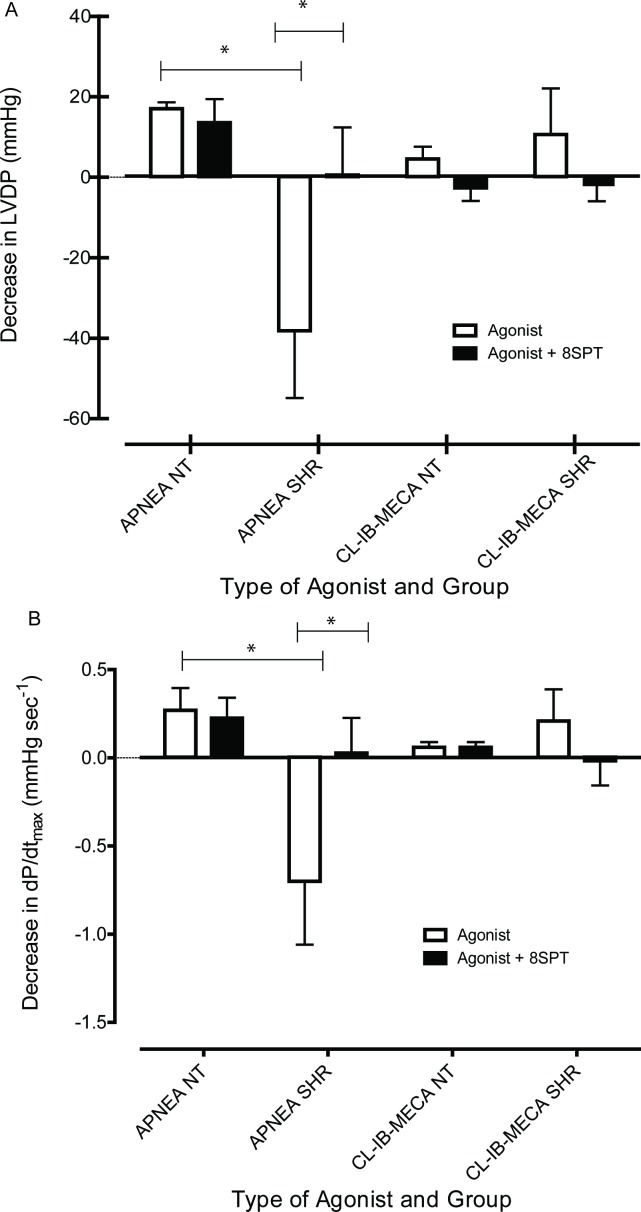
Mean decrease in (A) left ventricular developed pressure (LVDP) and (B) the left ventricle contractility (dP/dt) maximum at the concentration of 10^-5^M of APNEA or CL-IB-MECA for control normotensive rats (NT) and spontaneously hypertensive rats (SHR). Data obtained from each group was statistically analysed using one-way ANOVA followed by Tukey’s Multiple Comparison Test/Bonferroni correction. *A p value ≤0.05 was considered statistically significant.

The effect of 8SPT on heart contractile responses to APNEA and CL-IB-MECA can be seen in **Fig 4**. With respect to APNEA (10^-5^M), left ventricular developed pressure (LVDP) decreased approximately 20mmHg in normotensive rats, however, in the presence of 8SPT, APNEA did not alter LVDP (p>0.05). In contrast, APNEA caused an approximately 40mmHg increase in LVDP in hearts from SHR (**Fig 4A)**, which was significantly different to the control group (p<0.05). However, this effect of APNEA on LVDP was completely blocked in the presence of 8SPT in hearts from SHR (p<0.05). There was also a significant difference between normotensive and hypertensive rats in relation to dP/dt_(max),_ in which at APNEA (10^-5^M) caused an increase in dP/dt_(max)_ in hearts from SHR without affecting contractility in hearts from normotensive rats (p<0.05). The addition of 8SPT caused no significant effect in control hearts. However, in hypertensive hearts, as with LVDP, 8SPT completely blocked effects of APNEA on dP/dt_(max)_ (p<0.05). In addition, the effect of CL-IB-MECA (10^-5^M) on LVDP and dP/dt_(max)_ displayed no significant difference between control Wistar and SHRs (p>0.05), see **Fig 4B**. There were also no alterations to effects of CL-IB-MECA (10^-5^M) on heart contractile responses in the presence of 8SPT (p>0.05). This suggests that contractile responses to APNEA in hearts from SHR may be mediated via adenosine A_1_ receptors as CL-IB-MECA has very low affinity for this adenosine receptor subtype.

## Discussion

The purpose of this study was to investigate alterations in the expression of adenosine A_3_ receptors between SHRs and Wistar rats in the left ventricle, atria, blood, kidney and a range of blood vessels, and to determine the functional role of the adenosine A_3_ receptor in modulating coronary artery vasodilation. Spontaneously hypertensive rats were developed in 1963 and have been used extensively in high blood pressure research [16]. Because of the numerous limitations for sample collection in human studies, animal models of high blood pressure have been used to study physiological and genetic variants that may contribute to the development of essential hypertension (EH). Animals can exhibit several characteristics similar to those observed in humans with EH with the advantage of reducing the heterogeneity of genetic studies, as confounding factors are a major challenge for human genetic studies. Additionally, animal models are superior for characterising structural and functional variants, and in particular for performing invasive measurements and mechanistic studies [21]. Unlike human studies, an animal model can be controlled for many environmental factors, hence the research can focus on the genetic causes of hypertension.

Spontaneously hypertensive rats (SHRs) have been developed from the Wistar strain, without any treatment to induce high blood. Hence, it has been commonly used in research to study EH [16]. SHR blood pressure increases with age. The systolic blood pressure in SHRs rises to 150mmHg after 4 weeks of age and will reach a constant level approximately 170mmHg after 10 weeks [22]. Another study reported hemodynamic observed a significant increase in mean arterial pressure (MAP) and total peripheral resistance (TPR) at age 80–120 days was reported [23]. Additionally, SHRs develop complications of hypertension such as cerebral haemorrhage, nephrosclerosis and myocardial lesions [24]. With respect to gender differences, the body weights of male hypertensive rats were slightly increased when compared to female hypertensive rats. Furthermore, the systolic blood pressure level in male SHRs is about 11mmHg higher than female SHRs at 11 weeks of age. On the contrary, the blood pressure and body weights were not significantly different between male and female normotensive rats [16]. In order to eliminate variability due to effects of the sexual hormone oestrogen, which is known to affect cardiovascular function, this study only used male animals at 10 weeks of age.

In the present study, the SHR at 10 weeks of age had a conscious systolic blood pressure of approximately 174mmHg compared to age-matched Wistar rat systolic blood pressure of 108mmHg. Chronic hypertension causes an increase of left ventricular mass and is an independent risk factor for cardiovascular events [25]. Echocardiography and heart weight to body weight ratio (HWR) are commonly used to determine the presence of left ventricle hypertrophy. Left ventricle hypertrophy has been reported to develop in SHRs by 4 weeks of age [26]. Heart weight to body weight ratio is associated with high blood pressure (systolic and diastolic) [27]. Similar outcomes were confirmed in the present study as the HWR was significantly greater in 10 week old SHRs when compared to Wistar rats. Baseline parameters such as body weight, heart weight, and systolic blood pressure were obtained before perfusion on the Langendorff apparatus. Parameters such as left ventricular developed pressure (LVDP), and ventricle maximum and minimum slope (dP/dt_max/min_) were acquired during perfusion in the absence and presence of the adenosine A_1_ and A_2_ receptor antagonist, 8SPT (See **Table 2**). Comparison of these parameters between control and hypertensive hearts found that the latter tended to have increased heart contractile responses (LVDP and dP/dt_max/min_) under basal conditions (p<0.05) (see **Table 2**). Systolic blood pressure was also significantly higher in hypertensive rats (p<0.05). These significant differences in parameters indicate that hearts obtained from the SHR strain had developed a hypertensive phenotype [28].

We observed a substantial reduction in the expression of adenosine A_3_ receptors in the left ventricle, atria, thoracic aorta, portal vein and retina of 10 week SHRs. However, the expression pattern displayed a tissue-specific pattern (**Fig 1**). Kidneys have been reported to have a significant role in blood pressure control via renin-angiotensin-aldosterone system and adenosine receptors are considered to be involved in kidney pathophysiology in the development of hypertension. However, the mRNA expression of adenosine A_3_ receptor was below detectable levels in the renal artery (data not shown). In the present study, the expression of adenosine A_3_ receptors from blood samples reflected mRNA expression in kidney tissues, and did not show any significant difference between control Wistar rats and SHRs (**Fig 1**). We observed a substantial increase in the expression of adenosine A_3_ receptors in the mesenteric vessels from SHRs. It has been reported that mesenteric artery remodelling is associated with essential hypertension in human and rodent models [29]. In the present study, we have demonstrated that alterations in adenosine A_3_ receptor expression might contribute to essential hypertension. These results reveal the complexities of location and function of the adenosine A_3_ receptors in the vasculature.

The splice variant of adenosine A_3_ receptor was detected in brain and retina. A previous study investigating splice variants of the dopamine receptor reported that mRNA expression of the splice variants was elevated in the brain of SHRs with a possible role in the development of hypertension [30]. In the present study, we confirmed the expression of the adenosine A_3_ receptor splice variant was higher in the brain of SHRs compared to Wistar rats (data not shown), while there were no differences in the retina. Further study is warranted to characterise the splice variants of the adenosine A_3_ receptor and to determine whether it has a pathophysiological role in EH.

**Fig 5:**
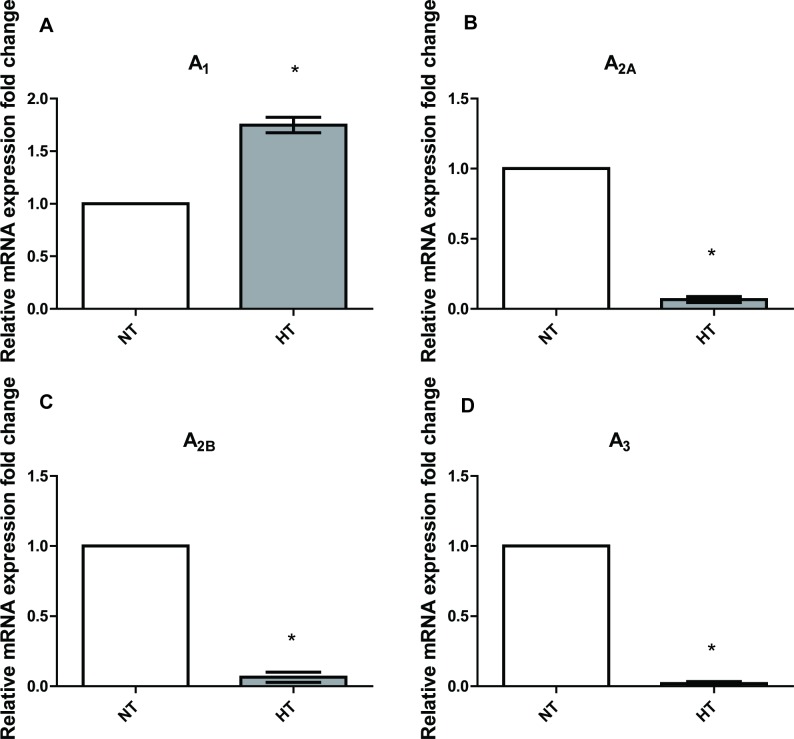
The relative expression of adenosine receptor subtype mRNA in left ventricle. A Student’s *t* test was performed as compared spontaneously hypertensive rat group (HT) with normotensive group (NT), (n = 8 for each group), *A p value ≤0.05 was considered statistically significant.

The expression of adenosine A_3_ receptor was down-regulated in the hearts of SHRs. We therefore set out to investigate the pharmacological effects of adenosine A_3_ receptor agonists on coronary vasodilation using the Langendorff heart perfusion preparation. Hearts were taken from a hypertensive model, in this case the SHRs, in addition to age-matched Wistar rats, which acted as the control. The Wistar rat is used as the control group as it is the strain from which the SHR rat is derived [31]. Hearts were prepared as outlined in **Methods**. The next series of functional experiments was to describe effects of APNEA, CL-IB-MECA and 8SPT on coronary adenosine A_3_ receptors. Isolation of the adenosine A_3_ receptor was achieved through the use of the adenosine A_1_ receptor and the adenosine A_2_ receptor antagonist, 8SPT (10^-5^M), and mediated effects were observed through the use of varying concentrations of either the partially selective adenosine A_3_ receptor agonist, APNEA, or selective the adenosine A_3_ receptor agonist, CL-IB-MECA. The adenosine A_3_ receptor has been reported to be present in rat aortic smooth muscle while pharmacological investigations have provided evidence for its presence in the coronary vessels of guinea pigs [32]. The adenosine A_3_ receptor has been reported to cause vasodilation in the heart, which has been achieved through the use of APNEA and CL-IB-MECA [33]. Concentration response curves of APNEA and CL-IB-MECA were performed in the absence and presence of the antagonist, 8SPT (10^-5^M). Perfusion of hearts with 8SPT present in Krebs-Hensleit solution, which allowed the blockade of the adenosine A_1_ receptor and adenosine A_2_ receptor induced vasodilation, adenosine A_1_ receptor mediated bradycardia, and isolation of the adenosine A_3_ receptor. Fozard and Carruthers [34] first demonstrated the use of 8SPT to isolate the adenosine A_3_ receptor, showing a decrease in blood pressure despite the presence of 8SPT, and thus concluded that it was unlikely to be mediated by the adenosine A_1_ receptor or the adenosine A_2_ receptor [34]. The discovery of the adenosine A_3_ receptor in rats by Zhou et al [35] provided an explanation for the drop in blood pressure, and thus the observations of Fozard and Carruthers have been attributed to the adenosine A_3_ receptor, although 8SPT is also able to block the adenosine A_1_ receptor mediated bradycardia, which alternatively could alter coronary flow rates and pressures [34]. Coronary vascular smooth muscle relaxant responses to both APNEA and CL-IB-MECA were observed in control hearts in the absence of 8SPT. However, the coronary vasodilator responses to adenosine A_3_ agonists in SHR hearts were significantly decreased compared to control tissues. In the presence of 8SPT, a rightwards shift in the curve and vasodilator response was observed in control hearts. However, in hypertensive hearts, neither APNEA nor CL-IB-MECA was able to elicit a relaxant response, which might be associated with down-regulation of the adenosine A_3_ receptor in the heart (**Fig 1**). While a decrease in the adenosine A_3_ receptor expression was found, an increase in mRNA expression of the adenosine A_1_ receptor was observed in SHR hearts (**Fig 5**), thus the vasodilator response observed in the absence of 8SPT was thought to possibly be adenosine A_1_ receptor mediated.

In addition to the relaxant responses, an unexpected vasoconstrictor effect was also observed to precede vasodilation in the presence of 8SPT in both control and hypertensive hearts. Vasoconstriction has also been recognised to be caused by the adenosine A_1_ receptor in the coronary vessels, counteracting the adenosine A_2_ receptor mediated vasodilator response [9]. However, as the vasoconstrictor response was observed during perfusion with 8SPT, the adenosine A_1_ receptors would presumably be blocked. Therefore, the effect is unlikely due to this receptor subtype. There has been some evidence of vasoconstriction caused indirectly by the adenosine A_3_ receptors via mast cell activation to cause the release of histamine and thromboxane in peripheral tissues [9]. However, histamine mediated effects are primarily that of vasodilation whilst thromboxanes are known to be released from platelets. As this experimental protocol utilizes physiological salt solutions to perfuse the hearts, these events are unlikely due to lack of blood circulating [36]. Other studies have found that deletions of the adenosine A_3_ receptor gene were associated with a higher degree of coronary vasodilation, suggesting that the adenosine A_3_ receptor may additionally have a role in regulating vascular tone through its inhibitory nature via the G_i_ protein to cause decreases in cellular cAMP in smooth muscle [9].

A significant increase in LVDP and dP/dt_(max)_ during perfusion with APNEA occurred in the absence of 8SPT in hypertensive hearts, which was not observed in the control hearts. These effects were completely blocked in the presence of 8SPT in hypertensive hearts. As 8SPT was able to block this mediated increase in contractility, the effect may be elicited by the adenosine A_1_ receptors or the adenosine A_2_ receptors. As mentioned previously, there was a significant increase in the adenosine A_1_ receptor expression in SHR hearts compared to control hearts. This receptor subtype, is reported to mediate negative inotropic effects, however this research may indicate adenosine A_1_ receptors in a hypertensive model may mediate an increase in cardiac contractility.

Furthermore, decreases in the adenosine A_2A_ receptor and the adenosine A_2B_ receptor subtypes, in addition to the adenosine A_3_ receptor had also been shown to decrease in the hypertensive heart compared to control (**Fig 5**). While adenosine A_2A_ receptor subtype is not particularly well known to cause an increase in contractility, this occurrence has been previously reported [37], showing that increased contractility could be mediated by the adenosine A_2A_ receptor in ventricular myocytes from rats, although not as potently as the β1-adrenoceptor mediated stimulation. It has also been reported to occur in avian embryo ventricular myocytes [38]. Cardiac contraction is achieved through an increase in intracellular Ca^2+^ levels and protein kinase A and protein kinase C pathways [37]. There is, however, controversy over the ability of the adenosine A_2A_ receptor to have a positive inotropic effect due to a number of studies revealing otherwise, such as Willems and Headrick [39], who reported that the adenosine A_2A_ receptor caused no contractile effects in mouse hearts. Reports of the selective the adenosine A_2A_ receptor agonist, 2-[p-(2-carboxyethyl)-phenethylamino]-5'-N-ethylcarboxamido adenosine (CGS-21680) causing increased LVDP and coronary flow were suggested to be instead due to the Gregg phenomenon in which increased flow increases ventricular function [40]. Our results demonstrate that the coronary flow rates are similar between groups and greater vasodilator responses were observed in control hearts. Chandrasekera et al [41] also reported no contractile effect but instead a strong vasodilator response. They did however observe increases in contractility when testing the adenosine A_2B_ receptor although this finding has limited support [41]. The reported findings of increased contractility or lack of in the literature were all performed on myocytes obtained from healthy models, whereas the findings of increased contractility were observed in hypertensive rats, suggesting that more extensive research is required to not only determine the subtype responsible but differences in pathological state.

In summary, this study characterised the effect of adenosine A_3_ receptor on coronary vasodilation using the Langendorff isolated heart and determined changes in the adenosine A_3_ receptor with EH. These observations potentially open the way to pharmacologic manipulation of vasomotor activity by the use of adenosine A_3_ agonists.

## Supporting Information

S1 Appendix Reference gene stability, PCR amplification efficiency analysis and Primer sequences. (DOCX)

## Abbreviations

ADORs: Adenosine receptors
BMI: Body mass index
BP: Blood pressure
APNEA: N^6^-2-(4-aminophenyl)ethyladenosine
CI-IB-MECA: 2-chloro-N^6^-(3-iodobenzyl)adenosine-5'-N-methylcarboxamide
CGS 21680: 2-[p-(2-carboxyethyl)-phenethylamino]-5'-N-ethylcarboxamido adenosine
CPP: Coronary perfusion pressure
HWR: Heart weight to body weight ratio
LVDP: Left ventricular developed pressure
RAAS: Renin-angiotensin-aldosterone system
SHRs: Spontaneously hypertensive rats
HT: Hypertensive
NIBP: Non-invasive blood pressure
NT: Normotensive
MAP: Mean arterial pressure
TPR: Total peripheral resistance
