# Supplementary material for: Pharmacology of the Adenosine A3 Receptor in the Vasculature and Essential Hypertension
Source: PLoS One. 2016 Feb 23;11(2):e0150021. doi: 10.1371/journal.pone.0150021 (PMC4764345; doi:10.1371/journal.pone.0150021)
Supplement: S1 Appendix — (DOCX) [file pone.0150021.s001.docx]

**S1 Appendix.**

**Reference gene stability analysis**

A panel of 11 reference genes (18s, ACTB, Tfrc, Pgkl, Hprtl, Gusb, Tbp, Rpll3a, Gapd, B2M, and Ppia) was analysed in each rat tissue types studied for their relative expression stabilities. The geNorm module in qbase plus software ranks candidate reference genes based on the gene stability measure (M value), which compares the average pairwise variation of a given reference gene against values derived from the other reference genes. The reference gene with the highest M value indicates the lowest stability of the expression and vice versa. An high stability of expression was observed in tissues in the present study as M values averaged ≤ 0.2. The optimal number of reference targets in this experimental situation was 2 and the most stable constitutively expressed reference genes were 18S and ACTB. When the same calculation was done with the NormFinder software and 18S and ACTB were still ranked in top positions in terms of gene expression stability. Therefore, these two housekeeping genes were chosen to normalise Ct values for ADOR subtypes.

**PCR amplification efficiency analysis**

The primer efficiency was optimised by a forward and reverse primer concentration gradient from 0.6mM to 1.4mM. The amplification efficiency was measured using 5-fold serial dilution (1/20, 1/100, 1/500, 1/2500, 1/12500, and 1/62500) of cDNA template in duplicated measurement. The exponential amplification efficiency (E) value should be approximately 2. This value is calculated using the equation: E= 10^(-1/slope)^. If the slope is -3.32, the PCR reaction will reach 100% efficiency, as the PCR product exactly doubles at each cycle.

**E= 10^(-1/-3.32)^ = 2 = 100% efficiency**

The amplification efficiency of the gene of interest and the reference gene should be within 10% of each other ([257](#_ENREF_257)). The amplification efficiencies of 18S rRNA and ACTB were 1.95 and 1.96 respectively (r^2^=0.99). Additionally, the amplification efficiencies for the ADOR were approximately 2.0 (r^2^=0.99). The efficiency values for the reference genes need to be similar to the efficiency values for the genes of interest in order to meet the assumptions of the 2^-ΔΔCt^ method. Our results indicated that the efficiency between reference genes and ADOR subtypes were similar. Therefore, the assumption of PCR efficiency was met and 2^-ΔΔCt^ approach was a suitable method to analyse the real time PCR data.

**Primer sequences**

| Genebank  Accession number | Primer | Sequence 5’- 3’ | Annealing temperature | PCR size | Primer efficiency |
| --- | --- | --- | --- | --- | --- |
| NM_001025002.1 | r18s Fw | cttagagggacaagtggcg | 60℃ | 71bp | 1.95 |
|  | r18s Rev | ggacatctaagggcatcaca | 60℃ |  |  |
| NM_031144.3 | rACTB Fw | CACACTGTGCCCATCTATGA | 60℃ | 272bp | 1.96 |
|  | rACTB Rev | CCGATAGTGATGACCTGACC | 60℃ |  |  |
| NM_017115.2 | rA_1_Fw | GTTGGCGCCCTGGTCATCC | 60℃ | 195bp | 2.0 |
|  | rA_1_ Rev | CCGCTGGGTCACCACTGTC | 60℃ |  |  |
| NM_053294 | rA_2A_Fw | GGCTTGGTGACAGGTGTGAG | 60℃ | 122bp | 2.0 |
|  | rA_2A_ Rev | CGCAGGTCTTCGTGGAGTTC | 60℃ |  |  |
| NM_017161.1 | rA_2B_ Fw | GATCATCGCTGTCCTCTG | 60℃ | 239bp | 1.97 |
|  | rA_2B_ Rev | AGGAAGGACACACCCAAA | 60℃ |  |  |
| NM_012896.2 | rA_3_ Fw | TACCATGACAGATAAAGAGCCAG | 60℃ | 83bp | 1.99 |
|  | rA_3_ Rev | GCAAGAATGGCTGTTAAGTCCTT | 60℃ |  |  |
| NM_012896.2 | rA_3SV_ Fw | TGCTGGCCATTGCTGTAGACC | 60℃ | 90bp | 2.0 |
|  | rA_3SV_ Rev | GGGAGGAAAACGGTAAGTTCAC | 60℃ |  |  |
